# The Phenotype Paradox: Lessons From Natural Transcriptome Evolution on How to Engineer Plants

**DOI:** 10.3389/fpls.2020.00075

**Published:** 2020-02-18

**Authors:** Justin Law, Kangbo Ng, Oliver P. F. Windram

**Affiliations:** ^1^ Grand Challenges in Ecosystems and the Environment, Imperial College London, Ascot, United Kingdom; ^2^ The Francis Crick Institute, London, United Kingdom; ^3^ Institute for the Physics of Living Systems, University College London, London, United Kingdom

**Keywords:** directed evolution, gene network analysis, synthetic biology, systems biology, breeding and genomics

## Abstract

Plants have evolved genome complexity through iterative rounds of single gene and whole genome duplication. This has led to substantial expansion in transcription factor numbers following preferential retention and subsequent functional divergence of these regulatory genes. Here we review how this simple evolutionary network rewiring process, regulatory gene duplication followed by functional divergence, can be used to inspire synthetic biology approaches that seek to develop novel phenotypic variation for future trait based breeding programs in plants.

## Introduction

Single nucleotide variants are amongst the most prevalent modifications in genomes (Altshuler et al., 2010). Furthermore, classical genetics focuses on the use of non-synonymous/synonymous mutation rate ratios to infer a baseline level of selection on gene sequences. Whilst this can be useful to infer how protein sequence variants may contribute to phenotypes it remains incredibly challenging to infer how point mutations might give rise to novel phenotypes that are a culmination of the coordinated action of tens to thousands of genes. In 1973 Susumu Ohno (Ohno, 1970) suggested how gene duplication might help drive the evolution of new phenotypes. He reasoned that purifying selection acting on essential genes could be circumvented by sequence duplication allowing evolution of redundant protein sequences, giving rise to novel functionality. However, Ohno also recognised that novel phenotypes could also simply be achieved through evolution at regulatory sites in duplicate gene sequences. This could lead to altered spatiotemporal expression facilitating evolution of novel traits. This might help to overcome the negative impact of gene dosage effects, where increasing protein abundance destabilizes networks or pathways (Voordeckers et al., 2015). This premise also extends beyond a single gene, to multi gene, and whole genome duplication (WGD). Here duplicate cellular pathways or metabolic processes are free to evolve along different spatiotemporal expression trajectories. Thus, diversity and cellular plasticity are attained purely through differential regulation of duplicate gene sets. This could occur through sequential evolution of cistronic transcription factor binding sites (TFBS) that bring target genes under coordinated regulatory control, as may have been the case for certain metabolic pathways in plants (Shoji, 2019). Two paralogs, QPT1 and QPT2 encode an enzyme involved in nicotinamide adenine dinucleotide and nicotine biosynthesis in tobacco. QPT1 is expressed at basal levels whilst QPT2 exhibits coordinated expression with nicotine biosynthesis genes. Furthermore, the promoter of QPT2 contains three sequence motifs that the ERF189, a positive regulator of nicotine biosynthesis, binds to *in vitro*. These three motifs provide graded positive activation of QPT2. Overall this suggests that TFBS bound by ERF189 evolved within the promoter of QPT2 facilitating its integration into the nicotine biosynthesis regulon (Shoji and Hashimoto, 2011). Alternatively, genes encoding transcription factors (TFs) might be duplicated with altered expression and/or functionality of TFs culminating in pleotropic regulatory cascades, thereby impacting entire pathways and cellular subsystems ultimately driving phenotype evolution.

Gene duplication could reduce selective pressure on redundant sequences allowing neutral evolutionary processes to generate novel phenotypic plasticity that might subsequently serve as an evolutionary advantage (Ohno, 1970; Wilson et al., 1977). In plants, gene duplicates experience a relatively relaxed period of selection before they are either silenced or take on novel, redundant or semi-redundant roles (Lynch and Conery, 2000; Blanc and Wolfe, 2004; Maere et al., 2005; Jiao et al., 2011). During this evolutionary filtering process it is noteworthy that regulatory genes are often preferentially retained whilst their paralogs often undergo gene expression divergence (Blanc and Wolfe, 2004; Maere et al., 2005). This highlights the role that gene duplication plays in driving transcriptome network evolution.

Contemporary evolutionary studies have understandably focused on prokaryotes with short generation times. Genome sequencing of bacterial strains grown under the same environmental conditions for over 50000 generations revealed how bacterial lineages gained mutations in regulatory genes allowing them to functionally diverge and occupy concurrent niches within a continuous culture (Plucain et al., 2014). Directed evolution in bacteria has identified solutions that modify gene expression, including TFs, with functionality arising from non-functional gene networks (Crameri et al., 1997; Yokobayashi et al., 2002). Additionally, synthetically rewiring TF networks in bacteria and yeast have generated novel phenotypes under stressful conditions (Isalan et al., 2008; Windram et al., 2017). This again suggests that TF gene expression evolution can aid in the generation of phenotypic novelty.

**Figure 1:**
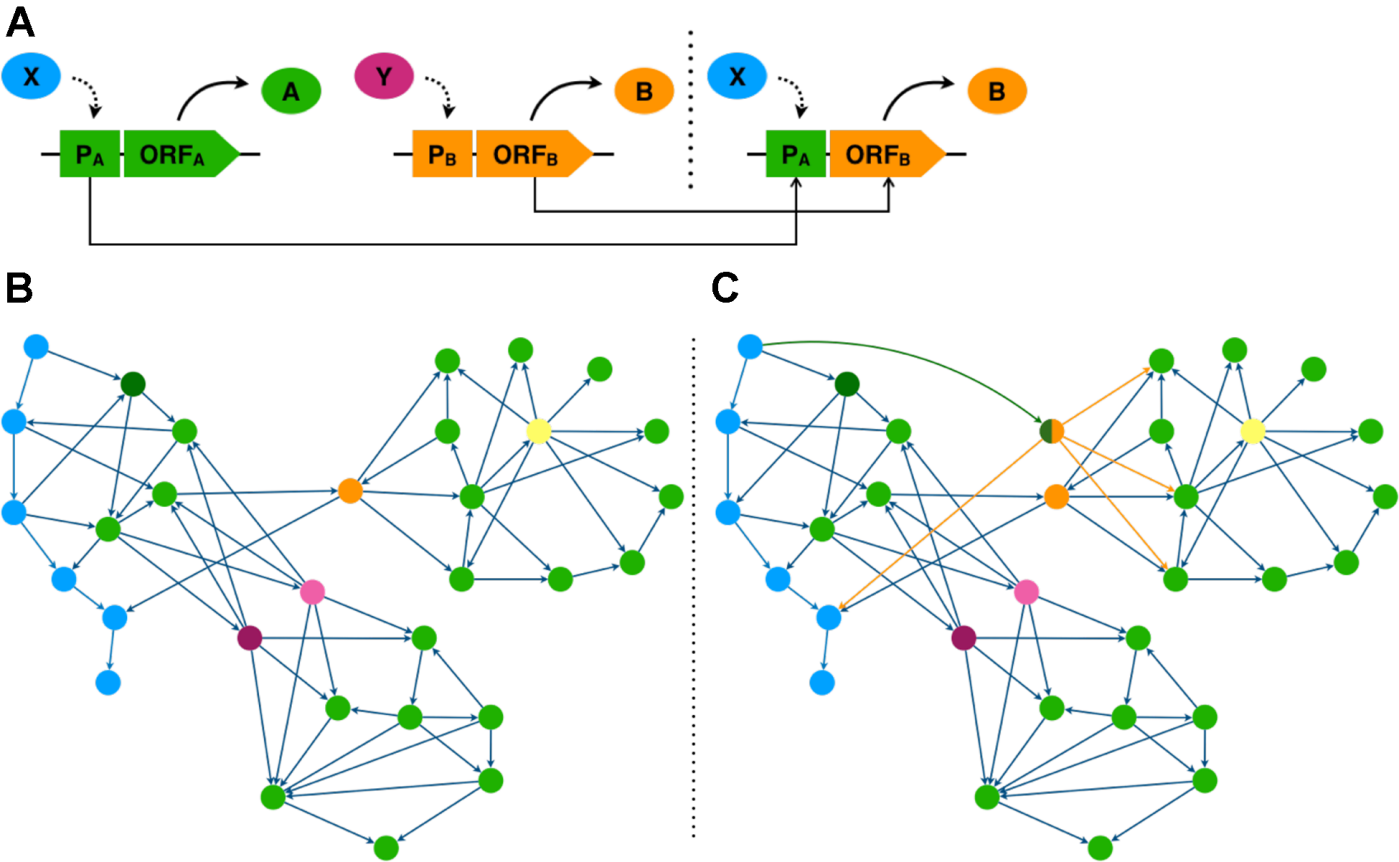
Transcriptional rewiring simulates TF evolution by gene duplication and expression divergence. **(A)** Promoter (P) region of TF gene A is fused to the open reading frame (ORF) of TF gene B generating a synthetic rewiring construct bringing ORF B under transcriptional control of TF X. **(B, C)** TF network diagrams, genes are represented as nodes and transcriptional regulation by edges. **(B)** native network. **(C)** rewired network taking the promoter region of the dark green gene and fusing it to the ORF of the orange gene. **(B, C)** genes coloured according to their network properties. Blue genes form a TF hierarchy. The orange gene has high betweenness centrality; the yellow gene, high out degree; and purple and pink genes are functionally redundant.

In this perspective we will highlight how evolution by gene duplication has shaped plant genomes. In particular, we will illustrate how evolution of duplicate TF gene expression, through modification of cistronic promoter sequences, helps to drive the generation of phenotypic novelty *via* cascading pleotropic regulation effects on target genes. Furthermore, we show how this process can be used to inspire the development of synthetic regulatory constructs that alter plant responses to environmental stress. We highlight how network structure can be used to select regulators for transcriptional rewiring (**Figure 1**). We show how this synthetic biology approach offers a novel way to optimise plant responses to environmental stimuli.

### Evidence for Transcriptional Rewiring Driving Plant Evolution and Domestication

Phylogenetic studies suggest that all flowering plants are palaeopolyploids having undergone at least two WGD events (Jiao et al., 2011). Although the fate of most duplicate genes is death by gene silencing (Lynch and Conery, 2000), it appears that transcriptional regulators are often preferentially retained, with some duplicates appearing to shape the developmental regulation that gave rise to seed bearing and flowering plants (Blanc and Wolfe, 2004; Maere et al., 2005; Jiao et al., 2011; Jiang et al., 2013). In Arabidopsis it seems that WGD drove TF numbers to increase by more than 90%. Duplicate gene expression rapidly diverges after these WGD events in some cases with entire, nonhomologous, co-regulated gene expression networks diverging alongside each other away from their cognate paralogs (Blanc and Wolfe, 2004). This coordinated divergence in expression of co-regulated genes suggests that upstream regulators may be undergoing evolution at the protein sequence or gene expression level culminating in altered expression of target genes. Furthermore, quantitative trait loci in promoters are selectively enriched within TFBS (Weirauch et al., 2014). Overall, this suggests that WGD and subsequent gene expression divergence drives functional divergence of gene duplicates.

Processes governing environmental stress response are known to involve complex transcriptional networks containing large TF families (Kreps et al., 2002; Windram et al., 2012; Lewis et al., 2015). These families have arisen through various forms of whole genome and single-gene duplication (Riechmann and Ratcliffe, 2000; Eulgem et al., 2000; Feller et al., 2011; Lehti-Shiu et al., 2017). Furthermore, genes involved in biotic stress response also appear to be preferentially retained after small scale and WGD events (Maere et al., 2005). It has also been noted that many historic WGD events in plants appear to have occurred during periods of major environmental stress and instability (Vanneste et al., 2014).

Gene duplication and expression divergence has influenced the genomes of many important crop species. For instance, gene duplication has shaped the evolution of metabolic pathways that affect the flavor and aroma of tea. Gene duplication has expanded gene families associated with synthesis of secondary metabolites in lipids, carotenoids, terpenoids, and shikimate, which serve as precursors to compounds that confer tea aroma and flavor, and gene families associated with the synthesis of catechins, which are responsible for the astringent taste found in tea (Wei et al., 2018). Gene duplication and subsequent expression divergence has also driven capsaicin biosynthesis evolution in peppers, where neofunctionalization of capsaicin synthase (CS), the enzyme responsible for the final step of capsaicin synthesis, occurred following a recent duplication event in peppers, which granted CS a role in capsaicinoid synthesis (Kim S. et al., 2014). Triads of homoeologs from wheat's three subgenomes exhibit striking relative expression differences across different tissue types (Ramírez-González et al., 2018). Thus it appears that expression bias within homeolog triads influences tissue specific transcriptome networks. Also, these dynamic triads were enriched for genes involved in defence, environmental responses and secondary metabolism. Swanson-Wagner and colleagues showed that maize co-expression networks have diverged significantly from maize's wild ancestor teosinte (Swanson-Wagner et al., 2012). Genes actively involved in this rewiring included TFs, while a number of genes involved in defence processes were differentially expressed between maize and teosinte. Similarly, differentially expressed paralogs in the seedlings of tomato and its wild relatives include genes involved in stress responses and defence responses (Koenig et al., 2013).

TF gene expression could evolve in several ways. Perhaps the most obvious is simple sequence perturbation *via* random point mutations within TFBS. This may have been how promoter evolution in a set of TF genes gave rise to both cold and drought tolerance in Arabidopsis (Haake et al., 2002). Also, a single nucleotide polymorphism in the regulatory region of the TF gene qSH1 is responsible for the loss of seed shattering during rice domestication (Konishi et al., 2006). Alternatively, random insertion of transposable elements (TE) might also significantly influence gene promoter activity. TE have been responsible for amplifying of E2F TFBS by 85% in Brassica species (Hénaff et al., 2014). TE also make up a substantial portion of many eukaryotic genomes (Wendel et al., 2016), up to 85% in the case of maize. TEs are often activated under periods of stress (Grandbastien, 1998) and appear to drive expression divergence in newly constructed synthetic wheat allotetraploids (Kashkush et al., 2002). Similarly, dynamically expressed homeologs in wheat more frequently contained transposable elements (TE) in their promoters and showed lower conservation of TFBS (Ramírez-González et al., 2018). Insertion of a TE into the regulatory region of the TF *teosinte branched1* (*tb1*) drives apical dominance in single stemmed maize by enhancing expression of *tb1* (Doebley et al., 1997; Studer et al., 2011).

Overall we see that WGD has significantly influenced the evolution of plant transcriptome networks. Whilst it has been observed that TF duplicates are more often retained after WGD rather than smaller duplication events (Maere et al., 2005), studies looking at domestication traits reveal a plethora of underlying single TFs with altered promoter sequences, appearing to drive TF expression divergence (Swinnen et al., 2016). This includes several TF genes with large TE element insertions in their regulatory regions suggesting that substantial regulatory rewiring can help to drive rapid TF expression divergence and trait evolution.

### Simulating Transcriptome Networks

Overall evolutionary studies suggest that TFs represent useful and logical targets for crop trait development using directed evolution. However, one major challenge is identification of key TFs to focus on. Plant genomes contain thousands of TFs, whilst several hundred might be involved in responses to an individual stimulus (Windram et al., 2012; Lewis et al., 2015). In this section we outline how modelling of transcriptome networks can be used to identify key transcriptional regulators in plant transcriptome networks.

There are many approaches to inferring gene regulatory networks (GRNs) from expression data (**Table 1**). Information theory based approaches (Zhang et al., 2012; Villaverde et al., 2014) use measures such as correlation and mutual information to establish relationships between genes. This approach is suitable for handling large amounts of expression data due to their relative simplicity and thus lower computational demands (Hecker et al., 2009), but application is limited to steady state data. The networks that are built using such approaches are typically undirected, meaning although relationships between genes are established, the regulator in these inferred interactions is unknown. The loss of this information is critical, as establishing the directionality of the relationships can give insight of how information flows through the network.

**Table 1:** Brief summary of the advantages and disadvantages of different network inference approaches.

Approach	Advantages	Disadvantages	Examples
Information Theory	Suitable for large number of genes low computational demand	Steady state expression input only Only considers pairwise comparisons	MIDER (Villaverde et al, 2014)
Dynamic Bayesian	Allows integration of prior knowledge Can handle noisy data	Computationally demanding	BACON (Godsey, 2013)
ODE	Can model different perturbations when system parameters are known Can handle steady state and time series data	Computationally demanding Functions chosen can limit interactions discovered Parameters difficult to determine	HiDi (Deng et al, 2017)
Machine Learning	Able to model complex relationships Can handle steady state and time series data	Computationally demanding Appropriate training data required	RegnANN (Grimaldi et al, 2011)

Table illustrates causal inference approaches are computationally demanding, which calls for the need for alternative solutions to improve the scalability of the algorithms. MIDER (Villaverde et al., 2014), BACON (Godsey, 2013), HiDi (Deng et al., 2017), RegnANN (Grimaldi et al., 2011).

To reconstruct directed networks, inference approaches such as Dynamic Bayesian Models, ordinary differential equation (ODE)-based models and machine learning-based models are often used (Delgado and Gómez-Vela, 2019). These approaches can take advantage of time series expression data to infer dynamical and causal relations between genes, with each having different limitations (**Table 1**). The selection of an appropriate approach to use is dependent on the biological system in question, and it has been demonstrated that combining predictions from different approaches produces better reconstructions of networks (Marbach et al., 2012). Although causal inference approaches are able to generate directed network models, a shared limitation in many state of the art algorithms is scalability. These approaches are typically very computationally intensive, and application is generally limited to small GRNs. Certain algorithms attempt to tackle this issue by using prior knowledge, for example the causal structural identification network inference method (Penfold et al., 2015) allows the selection of specific genes as potential TFs in order to reduce the number of computations. There have also been developments in algorithms specifically for large scale reconstructions (Thiagarajan et al., 2017; Liu et al., 2017), but their use has only been demonstrated on network sizes between 500 and 1,000 genes.

To interpret simple and complex networks, network measures can be calculated for each gene and examined. Network measures provide a numerical representation of how a gene controls information flow within the network, and so can often indicate the importance of a gene. Degree centrality is a measure of the number of interactions that a gene forms in a network. This can be separated into in-degree, the number of regulators a gene has, and out-degree, the number of target genes a TF gene has (**Figures 1B, C**). Key genes typically have high out-degree, as the higher number of target genes indicate greater regulatory influence, and are more likely to influence multiple biological processes (Jeong et al., 2001; Barabási and Oltvai, 2004; Yu et al., 2008). Betweenness centrality measures how often a gene mediates the shortest path between other gene pairs. High betweenness genes function as bridges between otherwise distant network modules. Thus the removal of such genes could severely disrupt information flow in the network (Yu et al., 2007) (**Figures 1B, C**). Hierarchy can also reflect importance, as influential genes are more likely to occupy higher positions, where they can exert greater control over the network through regulation of downstream TFs which allows changes to propagate through the network (Bhardwaj et al., 2010) (**Figures 1B, C**). The use of these existing network inference methods and the development of new methods that can deal with both directionality and scalability can be used to identify genes key to certain biological processes.

### Engineering the Transcriptome Using Genetic Rewiring

In this final section we seek to outline how targeted experimental interventions can be used to develop novel phenotypes using genetic rewiring. Specifically, we suggest how TFs identified through network analysis serve as pragmatic targets for plant trait creation. One way to artificially engineer the transcriptome network is to introduce an expression modified TF duplicate to effectively rewire the network (**Figure 1**). To do this the ORF of a TF gene is fused to the promoter region of a second gene. This rewiring of regulation allows signals to flow differently through the network, altering the spatiotemporal expression of the rewired TF and potentially its target genes (Isalan et al., 2008) (**Figure 1**).

Experimental rewiring of transcriptional networks in bacteria and yeast have revealed rewiring solutions that allowed these organisms to adapt to stressful environments (Isalan et al., 2008; Windram et al., 2017). Furthermore, studies of regulatory networks in plants suggest that stress response networks may be less tightly controlled and less complex than developmental networks (Jin et al., 2015). These plant stress networks appear to have shorter regulatory paths and lower interconnectivity. Moreover, our previous studies in yeast (Windram et al., 2017) further suggests that synthetic network rewiring that shortens hierarchies through fusion of top tier hierarchy gene promoters to lower tier ORFs with high out-degree and/or high betweenness centrality generates rewired networks with enhanced stress response phenotypes. In plants, to make the stress regulatory networks more responsive, we could “flatten” the regulatory hierarchy to improve the responsiveness of stress networks. With knowledge gained from network analysis we can select plant promoters that are at the top of hierarchies, and TF ORF with high degree and betweenness centrality.

By rewiring networks through introducing synthetic promoter-ORF fusions, an outcome akin to neofunctionalization of duplicated genes can be achieved. That is, this synthetic fusion expresses a second ORF in addition to the native one, but the synthetic ORF is regulated differently in space and time due to having a different promoter. As such, in an applied context, engineering plant phenotypes using transcriptome rewiring could provide interesting solutions to improve plant stress response. Rewiring could bypass the limitations of engineering plant phenotypes using genetic knockouts and constitutive overexpression of genes. These methods might strongly perturb signal flow through the transcriptomic network. As many TFs in regulatory networks form cooperative assemblies (protein-protein-DNA) a strong perturbation in TF protein levels might interfere with these assemblies, impeding network function. Constitutive overexpression of TFs, may outcompete other regulatory proteins that bind to target gene promoters, or titrate out rare cofactors (Rydenfelt et al., 2014). Comparatively TF knockouts directly reduce connectivity of the regulatory network, TF absence might also prevent certain transcriptional assemblies being formed. This strong biasing/reduction in connectivity in the regulatory network might lead to a decreased range of effective stress responses (Mittler, 2006; Atkinson and Urwin, 2012).

In the *Arabidopsis* immune network, it has been shown that *wrky4* mutants have reduced susceptibility to the biotrophic bacterial pathogen *Pseudomonas syringae*, but an increased susceptibility to the necrotrophic fungal pathogen *Botrytis cinerea* (Lai et al., 2008). It has also been shown that although constitutive overexpression of *wrky31* in rice reduces susceptibility towards the fungus *Magnaporthe grisea*, it also reduces lateral root elongation and formation (Zhang et al., 2008). These examples highlight how gene knockout and overexpression can have both beneficial and deleterious effects under different conditions. Because rewiring allows fine manipulation of the spatiotemporal regulation within the network, directed engineering to improve the plant against a specific type of stress may be possible, without substantially compromising the tunability of the network to deal with other types of stress (Tsuda et al., 2009; Tsuda and Katagiri, 2010; Kim Y. et al., 2014).

## Conclusion

Plants have revealed the tremendous potential for TF duplication and expression divergence to drive phenotype evolution. Similarly, for thousands of years crop breeders have sought out phenotypes that enhance yield, with many of these traits driven by TF rewiring. Advances in genomics and systems biology now afford us with the tools to study plant transcriptomes in tremendous detail and early experimental rewiring reveals a commonality in TFs that make good rewiring targets. The fascinating and complex polyploid genomes of crops, such as wheat, demonstrate not only a tolerance to TF rewiring but also offer up multiple TF sequences that can be targeted to drive selective improvement of such crops to specific environmental stresses.

## Author Contributions

JL, KN and OW all wrote the manuscript. JL and KN contributed equally to this work.

## Funding

This work is was supported by the Natural Environment Research Council (NE/M018768/1).

## Conflict of Interest

The authors declare that the research was conducted in the absence of any commercial or financial relationships that could be construed as a potential conflict of interest.

## References

[B1] Altshuler D. L. Durbin R. M. Abecasis G. R. Bentley D. R. Chakravarti A. Clark A. G. (2010). A map of human genome variation from population-scale sequencing. Nature 467, 1061–1073. 10.1038/nature09534 20981092 PMC3042601

[B2] Atkinson N. J. Urwin P. E. (2012). The interaction of plant biotic and abiotic stresses: from genes to the field. J. Exp. Bot. 63, 3523–3543. 10.1093/jxb/ers100 22467407

[B3] Barabási A. L. Oltvai Z. N. (2004). Network biology: understanding the cell's functional organization. Nat. Rev. Genet. 5, 101–113. 10.1038/nrg1272 14735121

[B4] Bhardwaj N. Kim P. M. Gerstein M. B. (2010). Rewiring of transcriptional regulatory networks: hierarchy, rather than connectivity, better reflects the importance of regulators. Sci. Signal. 3, ra79. 10.1126/scisignal.2001014 21045205

[B5] Blanc G. Wolfe K. H. (2004). Functional divergence of duplicated genes formed by polyploidy during arabidopsis evolution. Plant Cell 16, 1679–1691. 10.1105/tpc.021410 15208398 PMC514153

[B6] Crameri A. Dawes G. Rodriguez E. Silver S. Stemmer W. P. C. (1997). Molecular evolution of an arsenate detoxification pathway by dna shuffling. Nat. Biotechnol. 15, 436–438. 10.1038/nbt0597-436 9131621

[B7] Delgado F. M. Gómez-Vela F. (2019). Computational methods for gene regulatory networks reconstruction and analysis: a review. Artif. Intell. Med. 95, 133–145. 10.1016/j.artmed.2018.10.006 30420244

[B8] Deng Y. Zenil H. Tegnér J. Kiani N. A. (2017). HiDi: an efficient reverse engineering schema for large-scale dynamic regulatory network reconstruction using adaptive differentiation. Bioinformatics 33, 3964–3972. 10.1093/bioinformatics/btx501 28961895

[B9] Doebley J. Stec A. Hubbard L. (1997). The evolution of apical dominance in maize. Nature 386, 485–488. 10.1038/386485a0 9087405

[B10] Eulgem T. Rushton P. J. Robatzek S. Somssich I. E. (2000). The WRKY superfamily of plant transcription factors. Trends Plant Sci. 5, 199–206. 10.1016/S1360-1385(00)01600-9 10785665

[B11] Feller A. Machemer K. Braun E. L. Grotewold E. (2011). Evolutionary and comparative analysis of MYB and bHLH plant transcription factors. Plant J. 66, 94–116. 10.1111/j.1365-313X.2010.04459.x 21443626

[B12] Godsey B. (2013). Improved inference of gene regulatory networks through integrated Bayesian clustering and dynamic modeling of time-course expression data. PloS One 8. 10.1371/journal.pone.0068358 PMC3720774 23935862

[B13] Grandbastien M. A. (1998). Activation of plant retrotransposons under stress conditions. Trends Plant Sci. 3, 181–187. 10.1016/S1360-1385(98)01232-1

[B14] Grimaldi M. Visintainer R. Jurman G. (2011). Regnann: reverse engineering gene networks using artificial neural networks. PloS One 6. 10.1371/journal.pone.0028646 PMC3247226 22216103

[B15] Hénaff E. Vives C. Desvoyes B. Chaurasia A. Payet J. Gutierrez C. (2014). Extensive amplification of the E2F transcription factor binding sites by transposons during evolution of *Brassica* species. Plant J. 77, 852–862. 10.1111/tpj.12434 24447172

[B16] Haake V. Cook D. Riechmann J. L. Pineda O. Thomashow M. F. Zhang J. Z. (2002). Transcription factor CBF4 is a regulator of drought adaptation in Arabidopsis. Plant Physiol. 130, 639–648. 10.1104/pp.006478 12376631 PMC166593

[B17] Hecker M. Lambeck S. Toepfer S. van Someren E. Guthke R. (2009). Gene regulatory network inference: data integration in dynamic models-A review. BioSystems 96, 86–103. 10.1016/j.biosystems.2008.12.004 19150482

[B18] Isalan M. Lemerle C. Michalodimitrakis K. Horn C. Beltrao P. Raineri E. (2008). Evolvability and hierarchy in rewired bacterial gene networks. Nature 452, 840–845. 10.1038/nature06847 18421347 PMC2666274

[B19] Jeong H. Mason S. P. Barabási A. L. Oltvai Z. N. (2001). Lethality and centrality in protein networks. Nature 411, 41–42. 10.1038/35075138 11333967

[B20] Jiang W. K. Liu Y. L. Xia E. H. Gao L. Z. (2013). Prevalent role of gene features in determining evolutionary fates of whole-genome duplication duplicated genes in flowering plants. Plant Physiol. 161, 1844–1861. 10.1104/pp.112.200147 23396833 PMC3613460

[B21] Jiao Y. Wickett N. J. Ayyampalayam S. Chanderbali A. S. Landherr L. Ralph P. E. (2011). Ancestral polyploidy in seed plants and angiosperms. Nature 473, 97–100. 10.1038/nature09916 21478875

[B22] Jin J. He K. Tang X. Li Z. Lv L. Zhao Y. (2015). An Arabidopsis transcriptional regulatory map reveals distinct functional and evolutionary features of novel transcription factors. Mol. Biol. Evol. 32, 1767–1773. 10.1093/molbev/msv058 25750178 PMC4476157

[B23] Kashkush K. Feldman M. Levy A. A. (2002). Gene loss, silencing and activation in a newly synthesized wheat allotetraploid. Genetics 160, 1651–1659. 11973318 10.1093/genetics/160.4.1651 PMC1462064

[B24] Kim S. Park M. Yeom S. I. Kim Y. M. Lee J. M. Lee H. A. (2014). Genome sequence of the hot pepper provides insights into the evolution of pungency in Capsicum species. Nat. Genet. 46, 270–278. 10.1038/ng.2877 24441736

[B25] Kim Y. Tsuda K. Igarashi D. Hillmer R. A. Sakakibara H. Myers C. L. (2014). Mechanisms underlying robustness and tunability in a plant immune signaling network. Cell Host Microbe 15, 84–94. 10.1016/j.chom.2013.12.002 24439900 PMC4075322

[B26] Koenig D. Jiménez-Gómez J. M. Kimura S. Fulop D. Chitwood D. H. Headland L. R. (2013). Comparative transcriptomics reveals patterns of selection in domesticated and wild tomato. Proc. Natl. Acad. Sci. U. S. A. 110, E2655–E2662. 10.1073/pnas.1309606110 23803858 PMC3710864

[B27] Konishi S. Izawa T. Lin S. Y. Ebana K. Fukuta Y. Sasaki T. (2006). An SNP caused loss of seed shattering during rice domestication. Sci. (80-. ) 312, 1392–1396. 10.1126/science.1126410 16614172

[B28] Kreps J. A. Wu Y. Chang H. S. Zhu T. Wang X. Harper J. F. (2002). Transcriptome changes for Arabidopsis in response to salt, osmotic, and cold stress. Plant Physiol. 130, 2129–2141. 10.1104/pp.008532 12481097 PMC166725

[B29] Lai Z. Vinod K. Zheng Z. Fan B. Chen Z. (2008). Roles of Arabidopsis WRKY3 and WRKY4 transcription factors in plant responses to pathogens. BMC Plant Biol. 8, 68. 10.1186/1471-2229-8-68 18570649 PMC2464603

[B30] Lehti-Shiu M. D. Panchy N. Wang P. Uygun S. Shiu S. H. (2017). Diversity, expansion, and evolutionary novelty of plant DNA-binding transcription factor families. Biochim. Biophys. Acta - Gene Regul. Mech. 1860, 3–20. 10.1016/j.bbagrm.2016.08.005 27522016

[B31] Lewis L. A. Polanski K. de Torres-Zabala M. Jayaraman S. Bowden L. Moore J. (2015). Transcriptional dynamics driving MAMP-triggered immunity and pathogen effector-mediated immunosuppression in Arabidopsis leaves following infection with Pseudomonas syringae pv tomato DC3000. Plant Cell 27, 3038–3064. 10.1105/tpc.15.00471 26566919 PMC4682296

[B32] Liu J. Chi Y. Zhu C. Jin Y. (2017). A time series driven decomposed evolutionary optimization approach for reconstructing large-scale gene regulatory networks based on fuzzy cognitive maps. BMC Bioinf. 18, 241. 10.1186/s12859-017-1657-1 PMC5423002 28482795

[B33] Lynch M. Conery J. S. (2000). The evolutionary fate and consequences of duplicate genes. Sci. (80-. ). 290, 1151–1155. 10.1126/science.290.5494.1151 11073452

[B34] Maere S. De Bodt S. Raes J. Casneuf T. Van Montagu M. Kuiper M. (2005). Modeling gene and genome duplications in eukaryotes. Proc. Natl. Acad. Sci. 102, 5454–5459. 10.1073/pnas.0501102102 15800040 PMC556253

[B35] Marbach D. Costello J. C. Küffner R. Vega N. M. Prill R. J. Camacho D. M. (2012). Wisdom of crowds for robust gene network inference. Nat. Methods 9, 796–804. 10.1038/nmeth.2016 22796662 PMC3512113

[B36] Mittler R. (2006). Abiotic stress, the field environment and stress combination. Trends Plant Sci. 11, 15–19. 10.1016/j.tplants.2005.11.002 16359910

[B37] Ohno S. (1970). Evolution by gene duplication (London: Allen & Unwin). 10.1007/978-3-642-86659-3

[B38] Penfold C. A. Shifaz A. Brown P. E. Nicholson A. Wild D. L. (2015). CSI: a nonparametric Bayesian approach to network inference from multiple perturbed time series gene expression data. Stat. Appl. Genet. Mol. Biol. 14, 307–310. 10.1515/sagmb-2014-0082 26030796

[B39] Plucain J. Hindré T. Le Gac M. Tenaillon O. Cruveiller S. Médigue C. (2014). Epistasis and allele specificity in the emergence of a stable polymorphism in Escherichia coli. Sci. (80-. ) 343, 1366–1369. 10.1126/science.1248688 24603152

[B40] Ramírez-González R. H. Borrill P. Lang D. Harrington S. A. Brinton J. Venturini L. (2018). The transcriptional landscape of polyploid wheat. Sci. (80-. ) 361, eaar6089. 10.1126/science.aar6089 30115782

[B41] Riechmann J. L. Ratcliffe O. J. (2000). A genomic perspective on plant transcription factors. Curr. Opin. Plant Biol. 3, 423–434. 10.1016/S1369-5266(00)00107-2 11019812

[B42] Rydenfelt M. Cox R. S. Garcia H. Phillips R. (2014). Statistical mechanical model of coupled transcription from multiple promoters due to transcription factor titration. Phys. Rev. E. Stat. Nonlin. Soft Matter Phys. 89, 012702. 10.1103/PhysRevE.89.012702 24580252 PMC4043999

[B43] Shoji T. Hashimoto T. (2011). Recruitment of a duplicated primary metabolism gene into the nicotine biosynthesis regulon in tobacco. Plant J. 67, 949–959. 10.1111/j.1365-313X.2011.04647.x 21605206

[B44] Shoji T. (2019). The recruitment model of metabolic evolution: jasmonate-responsive transcription factors and a conceptual model for the evolution of metabolic pathways. Front. Plant Sci. 10, 560. 10.3389/fpls.2019.00560 31156658 PMC6528166

[B45] Studer A. Zhao Q. Ross-Ibarra J. Doebley J. (2011). Identification of a functional transposon insertion in the maize domestication gene tb1. Nat. Genet. 43, 1160–1163. 10.1038/ng.942 21946354 PMC3686474

[B46] Swanson-Wagner R. Briskine R. Schaefer R. Hufford M. B. Ross-Ibarra J. Myers C. L. (2012). Reshaping of the maize transcriptome by domestication. Proc. Natl. Acad. Sci. U. S. A. 109, 11878–11883. 10.1073/pnas.1201961109 22753482 PMC3406829

[B47] Swinnen G. Goossens A. Pauwels L. (2016). Lessons from domestication: targeting cis-regulatory elements for crop improvement. Trends Plant Sci. 21, 506–515. 10.1016/j.tplants.2016.01.014 26876195

[B48] Thiagarajan R. Alavi A. Podichetty J. T. Bazil J. N. Beard D. A. (2017). The feasibility of genome-scale biological network inference using graphics processing units. Algorithms Mol. Biol. 12, 8. 10.1186/s13015-017-0100-5 28344638 PMC5360040

[B49] Tsuda K. Katagiri F. (2010). Comparing signaling mechanisms engaged in pattern-triggered and effector-triggered immunity. Curr. Opin. Plant Biol. 13, 459–465. 10.1016/j.pbi.2010.04.006 20471306

[B50] Tsuda K. Sato M. Stoddard T. Glazebrook J. Katagiri F. (2009). Network properties of robust immunity in plants. PloS Genet. 5, e1000772. 10.1371/journal.pgen.1000772 20011122 PMC2782137

[B51] Vanneste K. Maere S. Van de Peer Y. (2014). Tangled up in two: a burst of genome duplications at the end of the Cretaceous and the consequences for plant evolution. Philos. Trans. R. Soc B Biol. Sci. 369. 10.1098/rstb.2013.0353 PMC4071526 24958926

[B52] Villaverde A. F. Ross J. Morán F. Banga J. R. (2014). MIDER: network inference with mutual information distance and entropy reduction. PloS One 9. 10.1371/journal.pone.0096732 PMC4013075 24806471

[B53] Voordeckers K. Pougach K. Verstrepen K. J. (2015). How do regulatory networks evolve and expand throughout evolution? Curr. Opin. Biotechnol. 34, 180–188. 10.1016/j.copbio.2015.02.001 25723843

[B54] Wei C. Yang H. Wang S. Zhao J. Liu C. Gao L. (2018). Draft genome sequence of Camellia sinensis var. sinensis provides insights into the evolution of the tea genome and tea quality. Proc. Natl. Acad. Sci. U. S. A. 115, E4151–E4158. 10.1073/pnas.1719622115 29678829 PMC5939082

[B55] Weirauch M. T. Yang A. Albu M. Cote A. G. Montenegro-Montero A. Drewe P. (2014). Determination and inference of eukaryotic transcription factor sequence specificity. Cell 158, 1431–1443. 10.1016/j.cell.2014.08.009 25215497 PMC4163041

[B56] Wendel J. F. Jackson S. A. Meyers B. C. Wing R. A. (2016). Evolution of plant genome architecture. Genome Biol. 17. 10.1186/s13059-016-0908-1 PMC4772531 26926526

[B57] Wilson A. C. Carlson S. S. White T. J. (1977). Biochemical Evolution. Annu. Rev. Biochem. 46, 573–639. 10.1146/annurev.bi.46.070177.003041 409339

[B58] Windram O. Madhou P. Mchattie S. Hill C. Hickman R. Cooke E. (2012). Arabidopsis defense against Botrytis cinerea: Chronology and regulation deciphered by high-resolution temporal transcriptomic analysis. Plant Cell 24, 3530–3557. 10.1105/tpc.112.102046 23023172 PMC3480286

[B59] Windram O. P. F. Rodrigues R. T. L. Lee S. Haines M. Bayer T. S. (2017). Engineering microbial phenotypes through rewiring of genetic networks. Nucleic Acids Res. 45, 4984–4993. 10.1093/nar/gkx197 28369627 PMC5416768

[B60] Yokobayashi Y. Weiss R. Arnold F. H. (2002). Directed evolution of a genetic circuit. Proc. Natl. Acad. Sci. U. S. A. 99, 16587–16591. 10.1073/pnas.252535999 12451174 PMC139187

[B61] Yu H. Kim P. M. Sprecher E. Trifonov V. Gerstein M. (2007). The Importance of Bottlenecks in Protein Networks: Correlation with Gene Essentiality and Expression Dynamics. PloS Comput. Biol. 3, e59. 10.1371/journal.pcbi.0030059 17447836 PMC1853125

[B62] Yu H. Braun P. Yildirim M. A. Lemmens I. Venkatesan K. Sahalie J. (2008). High-quality binary protein interaction map of the yeast interactome network. Sci. (80-. ). 322, 104–110. 10.1126/science.1158684 PMC2746753 18719252

[B63] Zhang J. Peng Y. Guo Z. (2008). Constitutive expression of pathogen-inducible OsWRKY31 enhances disease resistance and affects root growth and auxin response in transgenic rice plants. Cell Res. 18, 508–521. 10.1038/cr.2007.104 18071364

[B64] Zhang X. Zhao X.-M. He K. Lu L. Cao Y. Liu J. (2012). Inferring gene regulatory networks from gene expression data by path consistency algorithm based on conditional mutual information. Bioinformatics 28, 98–104. 10.1093/bioinformatics/btr626 22088843

